# Development of Novel Nano-Sized Imine Complexes Using *Coriandrum sativum* Extract: Structural Elucidation, Non-Isothermal Kinetic Study, Theoretical Investigation and Pharmaceutical Applications

**DOI:** 10.3390/ijms241814259

**Published:** 2023-09-19

**Authors:** Shimaa Hosny, Randa F. Abd El-Baki, Zeinab H. Abd El-Wahab, Gamal A. Gouda, Mohammed S. Saddik, Ateyatallah Aljuhani, Ahmed M. Abu-Dief

**Affiliations:** 1 Department of Chemistry, Faculty of Science, New Valley University, Alkharga 72511, Egypt; randafouad_1974@sci.nvu.edu.eg; 2 Department of Chemistry, Faculty of Science (Girl’s), Al-Azhar University, Cairo 11754, Egypt; zhabdelwahab@azhar.edu.eg; 3 Department of Chemistry, Faculty of Science, Al-Azhar University, Assiut Branch, Assiut 71524, Egypt; ggouda73@azhar.edu.eg; 4 Department of Pharmaceutics and Clinical Pharmacy, Faculty of Pharmacy, Sohag University, P.O. Box 82524, Sohag 82524, Egypt; mohammed.sherif@pharm.sohag.edu.eg; 5 Chemistry Department, College of Science, Taibah University, Madinah 42353, Saudi Arabia; assjuhani@taibahu.edu.sa; 6 Chemistry Department, Faculty of Science, Sohag University, Sohag 82524, Egypt

**Keywords:** green synthesis, nanocomplexes, DFT, anticancer activity, antimicrobial activity, COVID-19, kinetic parameter

## Abstract

A new Schiff base (H_2_L) generated from sulfamethazine (SMT), as well as its novel micro- and nanocomplexes with Ni(II) and Cd(II) metal ions, have been synthesized. The proposed structures of all isolated solid compounds were identified with physicochemical, spectral, and thermal techniques. Molar conductance studies confirmed that the metal complexes are not electrolytic. The molecular geometry located at the central metal ion was found to be square planar for the NiL_2_ and tetrahedral for the CdL_2_ complexes. The kinetic and thermal parameters were obtained using the Coats and Redfern approach. *Coriandrum sativum* (CS) in ethanol was used to create the eco-friendly Ni and Cd nanocomplexes. The size of the obtained nanoparticles was examined using PXRD and TEM, and found to be in the sub-nano range (3.07–4.61 nm). Furthermore, the TEM micrograph demonstrated a uniform and homogeneous surface morphology. The chemistry of the prepared nanocomplexes was studied using TGA and TEM techniques. The effect of temperature on the prepared nanocomplexes’ size revealed a decrease in size by heating. Furthermore, the nanocomplexes’ antimicrobial and anticancer properties were evaluated. The outcomes demonstrated that the nanocomplexes exhibited better antimicrobial properties. Moreover, the antitumor results showed that after heating, the Ni nanocomplex exhibited a substantial antitumor activity (IC_50_ = 1.280 g/mL), which was higher than the activity of cis-platin (IC_50_ = 1.714 g/mL). Finally, molecular-docking studies were performed to understand the evaluated compounds’ ability to bind to methionine adenosyl-transferases (PDB ID: 5A19) in liver cancer and COVID-19 main protease (PDB ID: 6lu7) cell-proteins. The findings reveal that [NiL_2_]·1.5H_2_O_2_ has a higher binding energy of −37.5 kcal/mol with (PDB ID: 5A19) cell protein.

## 1. Introduction

Schiff bases are formed through the condensation of primary amines and carbonyl compounds to generate imines or azomethine groups [1]. Schiff bases are important in the synthesis of Schiff base complexes because these ligands have the ability to form stable compounds with metal ions [2]. In addition to this, Schiff bases and their complexes have a broad range of biological activities [3,4].

On the other hand, sulfonamides, generally referred to as sulfa medicines, were the first routinely used chemotherapeutics for treating and preventing bacterial infections in humans [5]. Due to their uses, a wide variety of families of organic and inorganic compounds are now being studied. One of the most common compounds is sulfonamide and its N-derivatives [6]. Numerous Schiff bases derived from sulfonamide have been synthesized and used as chelates in the synthesis of strong metal chelates. Sulfamethazine is a sulfa-based antibacterial drug used for treating livestock diseases [7]. Like other sulfonamides, sulfamethazine has been modified through the creation of Schiff bases [8] and metal complexes [9]. The creation of nano-sized composites results in the synthesis of new compounds with distinctive physical, chemical, and biological characteristics [10].

Because of the huge number of surface atoms relative to atoms, nanoparticles (1–100 nm) generated by nanotechnological processes exhibit various novel physicochemical features in comparison to macro-sized structures [11,12]. Many studies on the creation of nanoparticles, which have a wide range of uses, have lately been carried out, and significant findings have been obtained. Although the physical and chemical technologies employed in nanoparticle synthesis enable the manufacture of nanoparticles of any size in a short amount of time, their toxicity is high [13]. In order to obtain harmless types of nanoparticles, it is necessary to employ newer and more practical techniques

The green synthesis of nano-metal-complexes offers an alternative, effective, low-cost, and eco-friendly way to create nanoparticles with defined geometries [14,15]. Researchers have succeeded in creating different nanoparticles for this purpose through the use of plant extracts and microorganisms [16]. The manufacture of nanoparticles using plants has not received much attention, according to a review of the relevant scientific literature. The production of nanoparticles from plant extracts is faster than the microbial synthesis, and the resulting nanoparticles have a more stable structure [17].

In this article, we have synthesized two novel micro- and nanocomposites with a ligand derived from sulfamethazine and 2-hydroxy-1-naphthaldehyde. Numerous methods, including physicochemical and analytical tools, were applied to describe the produced Schiff base and its micro- and nanocomposites. The chemistry of the generated nano-composites was studied using TGA and TEM techniques. Also, computational investigations, such as DFT and molecular docking, were carried out on the prepared compounds. The kinetics and thermodynamic parameters for some thermal decomposition steps have been critically studied. Moreover, the in vitro antibacterial, antifungal, and anticancer activities of the prepared compounds were examined.

## 2. Results and Discussion

### 2.1. Physical, Microanalytical, and Molar Conductance Data

According to the complex’s molar conductance measurement, which was carried out in 10^−3^ M DMF solvent and was between 0.7 and 12 ohm^−1^ cm^2^ mol^−1^, the complexes under examination have a non-electrolytic nature. The microanalytical data revealed that all metal chelates were synthesized with a 1:2 (M:L) stoichiometry (Table 1).

### 2.2. ^1^H-NMR

The ^1^H NMR spectrum of the newly prepared H_2_L ligand was recorded in DMSO-d6 (Figure S1). The peaks appear at δ 2.21, δ 9.64, and δ 2.51 ppm, which may be assigned to the CH_3_, OH, and DMSO-d6 [18] protons. The proton of azomethine was detected at δ 8.58 ppm [18], confirming the production of the Schiff base ligand. A signal at δ 10.61 ppm is due to the proton of the NH group [19]. The signals recorded between δ 7.24 and δ 7.91 ppm are characteristic of the aromatic protons [20].

### 2.3. Infrared Spectra

#### 2.3.1. IR Spectroscopy of Metal Complexes

The chelation mode between the ligand and the metal ions may be established by comparing the complex IR spectra to those of the free ligand (Figure S2). The finding proves that the ligand behaves in a bidentate manner. The characteristic vibrational bands were identified at 3200, 3457, 1384, and 1156 cm^−1^ for the free H_2_L ligand, assigned to υ(OH), υ(NH), υ_as_(SO_2_), and υ_sy_(SO_2_) groups, respectively. The (HC=N) vibration of the ligand at 1633 cm^−1^ indicates the synthesis of the H_2_L ligand; however, in the complexes of Ni(II) and Cd(II), this peak migrated to 1602 and 1624 cm^−1^, due to its coordination [21]. In the Ni(II) and Cd(II) complexes, the phenolic (O-H) of the H_2_L vanished, demonstrating proton transfer during complex formation. The persistence of the υ(NH) at 3457, υ_as_(SO_2_) at 1384 cm^−1^, and υ_sy_(SO_2_) at 1156 cm^−1^ in the spectra of the complexes and their free ligand rules out the coordination through these groups [22]. Finally, the peaks observed for the metal chelates at 544–549 and 566–585 cm^−1^ were assigned to (metal–nitrogen) and (metal–oxygen), respectively. Moreover, the band at 970 in the spectrum of the Ni(II) chelate could be ascribed to the (H_2_O) [23] (Table S1).

#### 2.3.2. IR Spectroscopy of Nano-Complexes

The binding modes of the Ni and Cd nanocomposites produced in CS/EtOH media (Figure S3) are revealed by the FTIR spectra. This is corroborated by a shift in the peak location of the nanocomposites as compared to the free ligand.

### 2.4. Mass Spectra

**Figure 1:**
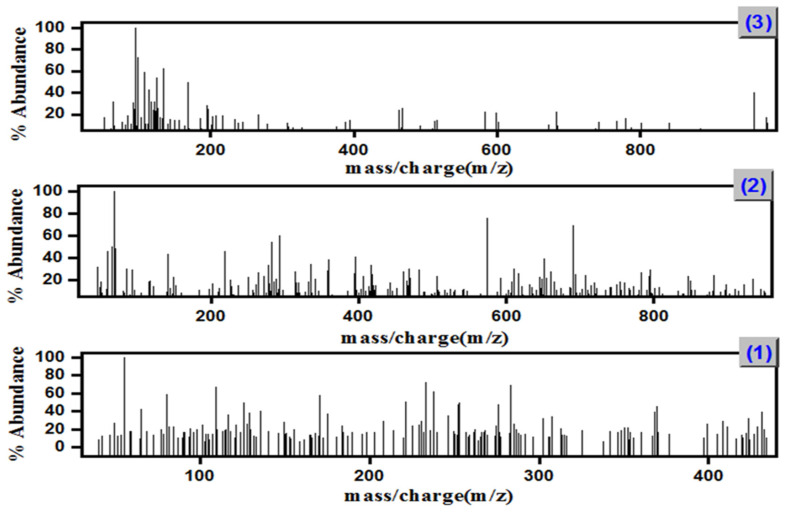
Mass spectra diagrams of (**1**) H_2_L ligand; (**2**) Ni(II) complex; and (**3**) Cd(II) complex.

Mass spectrometry has been used successfully to confirm the H_2_L Schiff base’s molecular ion peaks and its metal complexes. The observed mass spectrum of the H_2_L ligand indicates a molecular ion peak (M^+^) at 432.50, which strongly supports the proposed formula. The fragment pattern of the H_2_L ligand provides an impression of the possible deterioration, with a sequence of peaks corresponding to different fragments. The suggested fragmentation pattern of the H_2_L is given in a supplementary data file (Scheme S1). The MS of Ni(II) and Cd(II) complexes showed molecular ion peaks at 948.73 and 975.79, respectively, which suited the molecular weights of these complexes. They also showed that Ni and Cd isotope peaks were present at *m*/*z* 60 and 114, respectively (Figure 1).

### 2.5. XRD and Morphological Studies

#### 2.5.1. XRD of Nanocomplexes

**Figure 2:**
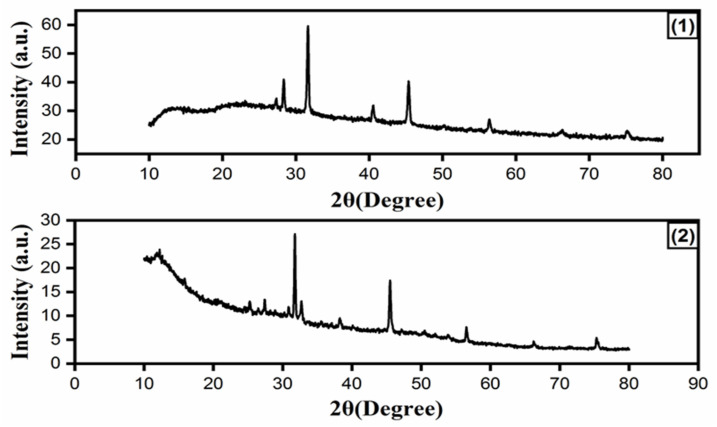
The XRD patterns of (**1**) Ni and (**2**) Cd nanocomplexes in CS media.

The obtained Ni and Cd nanocomplexes in CS/EtOH media were examined by the powder XRD method (Figure 2). XRD patterns suggested that the prepared nanocomplexes lie between amorphous and crystalline characters. As a result, it was found that nanocomplexes were unsuitable for single-crystal XRD. The peak broadening indicated that the prepared compounds are nanoscale in size [24]. The XRD pattern shows that the crystal size can be calculated according to the Debye–Scherrer formula [18,25]. The average crystal sizes of Ni and Cd nanocomplexes are 33.1 and 36.4 nm, respectively.

#### 2.5.2. TEM Analysis

**Figure 3:**
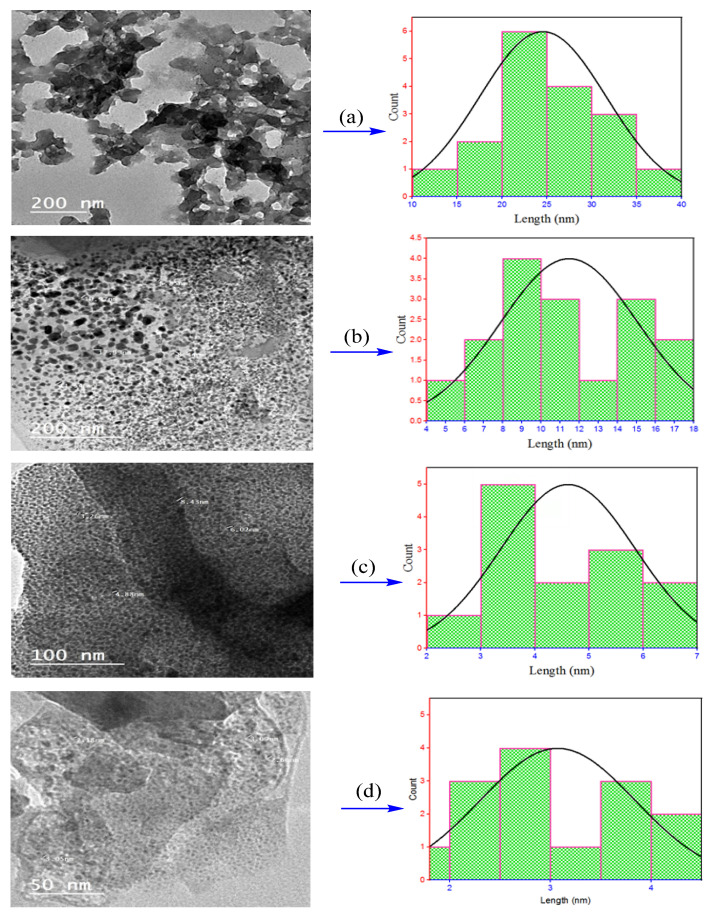
TEM images and histogram of particle size distribution for Ni and Cd nanocomplexes before heating (**a**,**b**) and after heating at 200 °C (**c**,**d**).

The morphology and size of Ni and Cd nanoparticles created in *Coriandrum sativum* (CS) media were studied, as well as the impact of heat on the prepared nanocomplexes after heating them at 200 °C for two hours using the TEM technique (Figure 3). The images demonstrate particle morphologies to be homogeneous and close together, which is evidence that there are identical matrices. The mean particle size of Ni and Cd nanocomplexes before heating was about 24.55 and 33.89 nm, respectively. However, after heating, the Ni and Cd nanocomposites at 200 °C were 4.61 and 3.07 nm (sub-nano), respectively, indicating that heating reduces the particle size of the formed nanocomplexes. These findings accord well with the estimated crystallite size values from the XRD technique.

### 2.6. Thermal Behavior

#### 2.6.1. TG-DTG of Complexes

**Figure 4:**
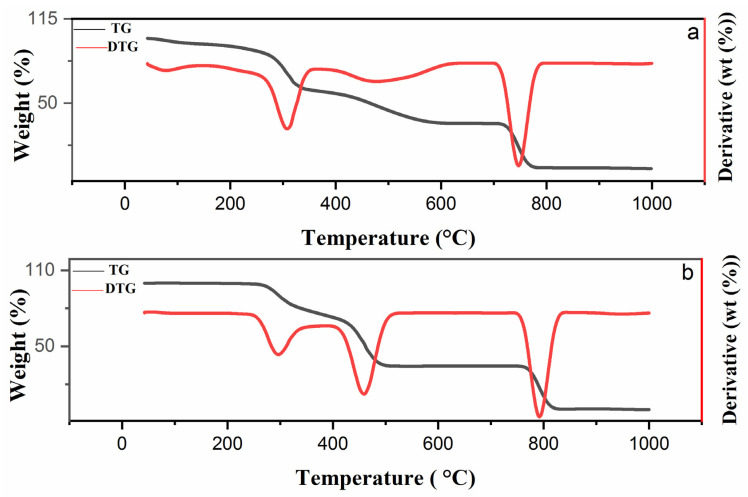
TG and DTG curves of the [NiL_2_]·1.5H_2_O (**a**) and CdL_2_ (**b**) complexes.

The TG-DTG thermograms of Ni(II) and Cd(II) chelates are shown in Figure 4. The TG curve of the Ni(II) chelate exposed four decomposition steps. The first step from 52 to 111 °C with an estimated mass loss of 2.32% (calc. 2.84%) corresponds to the removal of the non-coordinated H_2_O molecules. The second step, from 265 to 321 °C with a mass loss of 20.93% (calc. 22.58%), corresponds to the removal of 2C_6_H_8_N_2_. The third decomposition step occurred at temperatures ranging from 404 to 566 °C with a mass loss of 21.70% (calc. 22.79%), corresponding to the loss of SO_2_ and 2C_6_H_6_. At a higher temperature of 727–767 °C, a mass loss of 30.01% (calc. 30.18%) is attributed to the elimination of 2C_10_H_7_O. The TG curve of the Cd(II) chelate exposed a three-step decomposition. The Cd(II) complex showed no mass loss up to 271 °C, confirming the absence of coordinated water and the complex’s high thermal stability. The mass losses in temperature from 271 to 318 °C with a mass loss of 21.69% (calc. 21.96%) may be attributed to the decomposition of 2C_6_H_8_N_2_ followed by the loss of 2C_6_H_6_, SO_2_ and 2HCN with a mass loss of 26.19% (calc. 27.71%) within the temperature from 420 to 481 °C. After this decomposition, the mass loss of the third decomposition stage was within the range from 772 to 811 °C with a mass loss of 30.42% (calc. 29.14%), corresponding to the removal of the rest of 2C_10_H_7_O. In Ni(II) and Cd(II) chelates, metal oxides were left as a residue.

#### 2.6.2. TG-DTG of Nanocomplexes

**Figure 5:**
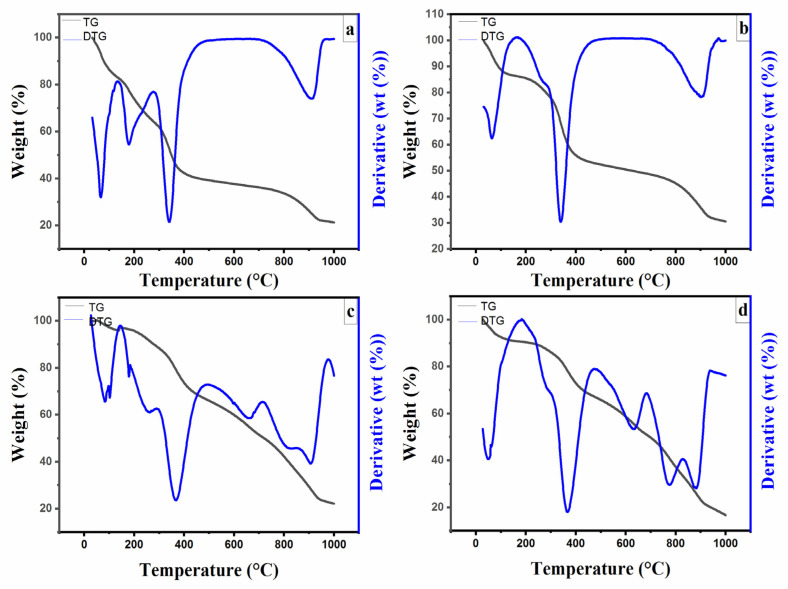
TG-DTG curves of Ni nanocomplex (**a**,**b**) and Cd nanocomplex (**c**,**d**) before and after heating, respectively.

The thermogram of nickel and cadmium nanodomain metal chelates prepared in *Coriandrum sativum* (CS) extract in 20% ethanol aims to discover the chemistry of nanodomain metal chelates by evaluating the size of the nanodomain metal chelates at each step of thermal heating to ascertain how the heat affects the size of the nanodomain metal chelates (Figure 5). The thermograms of Ni and Cd nanodomain were studied before and after heating the nanodomain chelates at 200 °C for 2 h. The mean particle size of Ni and Cd nano-chelates before heating was about 10.11–21.39 and 12.90–31.96 nm, respectively. However, after heating, the Ni and Cd nano-chelates at 200 °C were 2.05–39.6 nm (sub-nano) and 1.08–2.78 nm (sub-nano), respectively, indicating that heating reduces the particle size of the formed nano-chelates (Table 2 and Table 3).

#### 2.6.3. Kinetic Calculations

In the present work, the kinetic and thermodynamic parameters, the energy of activation (Ea), the activation enthalpy (ΔH*), the entropy of activation (ΔS*), the Gibbs energy change (ΔG*), the order of the reaction (n), the correlation coefficient (r), and the pre-exponential factor (Z) for the non-isothermal decomposition of the prepared metal complexes were determined by the integral method proposed by Coats and Redfern [26]. The obtained data are listed in Table 4. Using the following equations, the activation enthalpy (ΔH*), the activation entropy (ΔS*), and the free energy (ΔG*) were determined.

ΔS* = 2.303 [log (Zh/KT)]R

ΔH* = E − RT

ΔG* = ΔH − TS ΔS

**Figure 6:**
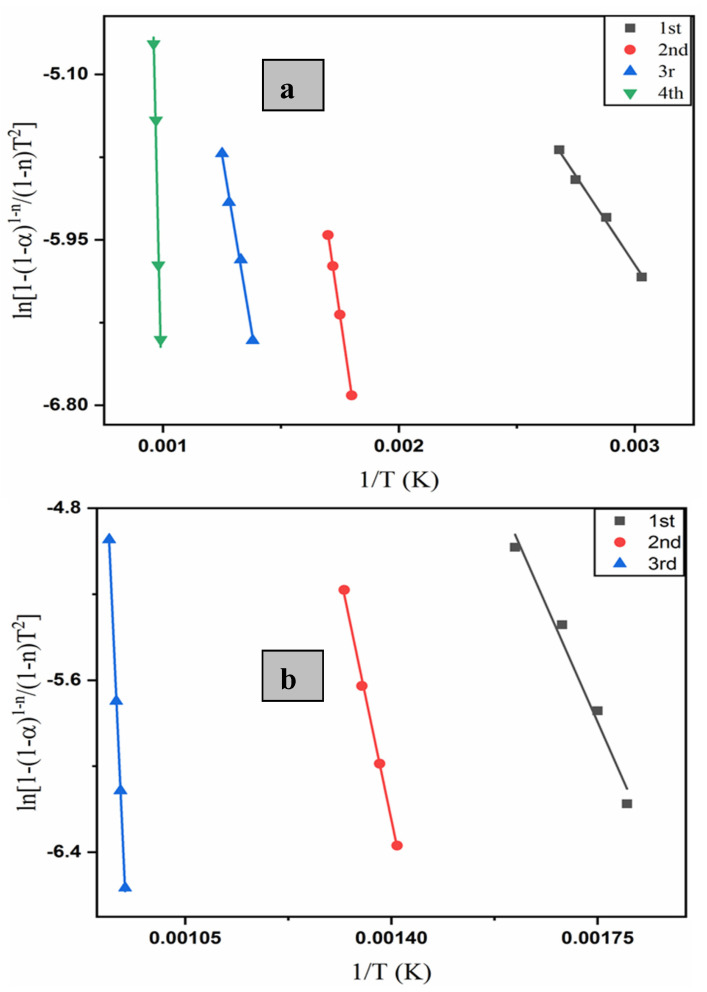
Coats–Redfern curves of Ni(II) complex (**a**) and Cd(II) complex (**b**).

The high E_a_ values illustrate that the H_2_L is powerfully bonded to the Ni(II) and Cd(II) ions. The existence of negative ΔS* values in NiL_2_ (first, second, and third steps) and CdL_2_ (first step) demonstrates that the activated complexes are more ordered and the reactants are slower [27]. The positive activation enthalpy ΔH* data refer to the endothermic decomposition process. The positive values of ΔG* mean that the decomposition reaction is not spontaneous [28]. Figure 6 shows the plots of ln[1(1 − α)1 − n/(1 − n)T^2^] vs. 1/T for different models of Ni(II) and Cd(II) chelates, respectively, demonstrating that all models exhibit a linear trend with a good correlation coefficient.

### 2.7. UV-Visible Absorption Study

The electronic spectra of the prepared compounds were noted in DMF (10^−4^ M) at room temperature. The free ligand demonstrated that absorption bands appear at 386, 445, and 468 nm due to the π→π* transition of the aromatic ring, n→π* of azomethine (HC=N), and n→π* of the phenolic group, respectively. Additionally, the band appearing at 539 nm may be due the charge-transfer (CT). The Ni(II) chelates exhibit three distinct bands at 365, 408, and 542 nm, attributed, respectively, to the transitions n→π* of azomethine (HC=N), ^1^A_1g_→^1^B_1g_, and ^1^A_1g_→^1^A_2g_ for the square planar Ni(II) chelate [29]. The electronic spectrum of the Cd(II) chelate demonstrated bands due to n→π* transition of azomethine (HC=N), which appeared at 456 nm. The second band is observed at 525 nm, which may be due to the CT transition [18,30]. The electronic spectrum of green-synthesized Ni and Cd nanocomplexes displayed a band related to the surface plasmon resonance (SPR) [31], proving the formation of nanocomposites. The Ni nanocomplexes exhibit bands at 363, 399, and 535 nm, attributed, respectively, to the transitions n→π* of imine (C=N), ^1^A_1g_→^1^B_1g_, and ^1^A_1g_→^1^A_2g_ for the square planar Ni nanocomplex. The spectra of the Cd nanocomplex show absorption bands at 406 nm which can be assigned to the imine (C=N) group. Finally, there is an absorption band at 513 nm, which may refer to a charge-transfer band caused by a change in electron distribution between the metal and a ligand. The spectra of Ni(II) and Cd(II) chelates and their nanocomplexes are shown in the supporting information (Figure S4), and the electronic spectral data are shown in Table 5.

### 2.8. Molecular Modeling

#### 2.8.1. Geometry Optimization of the H_2_L Ligand

**Figure 7:**
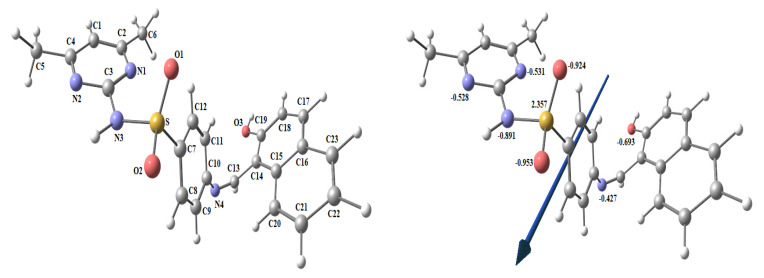
Geometry-optimized structure, dipole moment, and the charges on active centers of the H_2_L ligand.

The most appropriate structural configuration of the H_2_L ligand is shown in Figure 7. The charges from the natural bond orbital (NBO) analysis are (−0.693), (−0.953), (−0.924), (−0.531), (−0.528), (−0.891), (−0.427), and (2.357) for O1, O2, O3, N1, N2, N3, N4, and S, respectively. This supports the coordination of metal ions with O3 and N4 in a bidentate manner.

#### 2.8.2. Optimization of [NiL_2_]·1.5H_2_O

**Figure 8:**
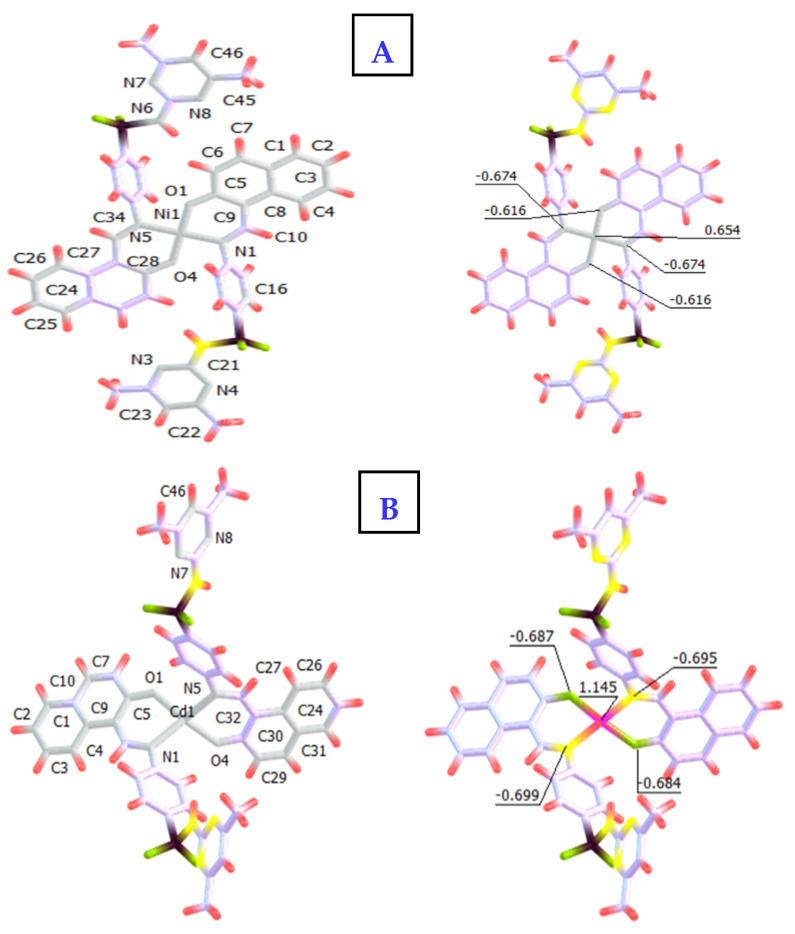
Geometry–optimized structures and the charges on active centers of [NiL_2_]·1.5H_2_O (**A**) and [CdL_2_] (**B**).

The appropriate structural configuration of the [NiL_2_]·1.5H_2_O (Figure 8A) was demonstrated to support the ligand and metal ion interaction preferences. The nickel atom is four-coordinated in a square planar geometry in which the atoms O1, N1, O4, and N5 deviate from the plane by 0.008° degrees (Table 6). The bond lengths between N1-----O1 and N5-----O4 in the ligand (3.715 Å) are reduced in the [NiL_2_]·1.5H_2_O to 2.690, as a result of coordination. Also, the charges of coordinating atoms were, Ni (+0.654), O1 (−0.616), O4 (−0.616), N1 (−0.674), and N5 (−0.674).

#### 2.8.3. Optimization of [CdL_2_]

The [CdL_2_] structural configuration (Figure 8B) was shown to support the ligand and metal ion interaction preferences. The cadmium atom is four-coordinated in a tetrahedral geometry (Table 6). The bond lengths between N1-----O1 and N5-----O4 in the ligand (3.715 Å) are reduced in the [CdL_2_] to 2.950 Å and 2.948 Å for N5-----O4, as a result of coordination. Also, the charges of coordinating atoms were Cd (+1.145), O1 (−0.695), O4 (−0.699), N1 (−0.684), and N5 (−0.687).

#### 2.8.4. Molecular Orbitals (MO) and MEP Maps

**Figure 9:**
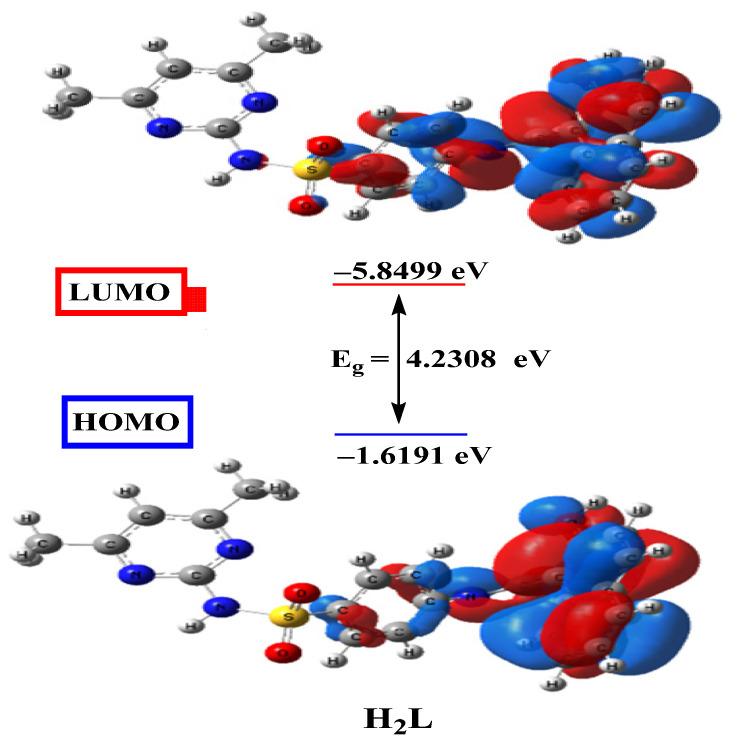
Surface plots of HOMO − LUMO of the H_2_L ligand.

**Figure 10:**
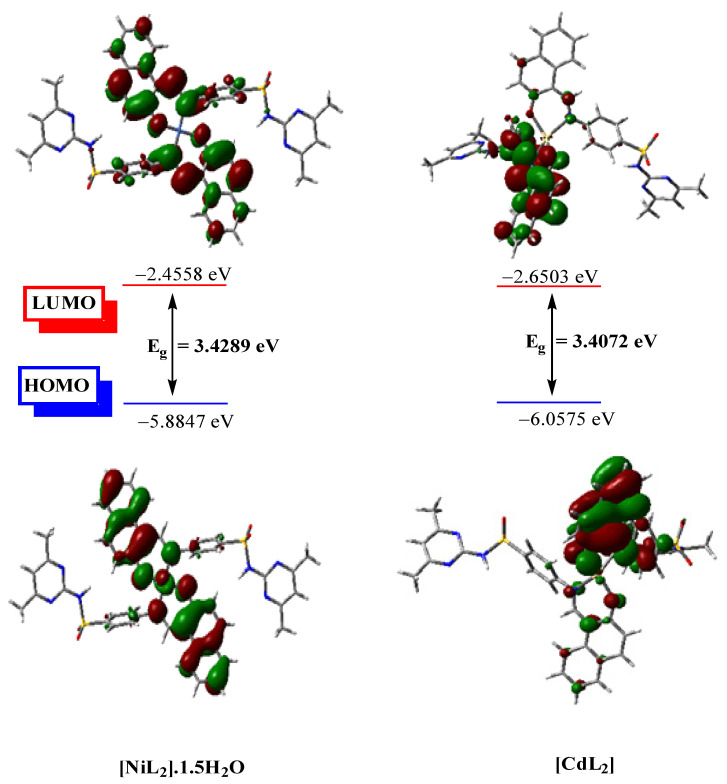
Surface plots of HOMO-LUMO of [NiL_2_]·1.5H_2_O and [CdL_2_].

**Figure 11:**
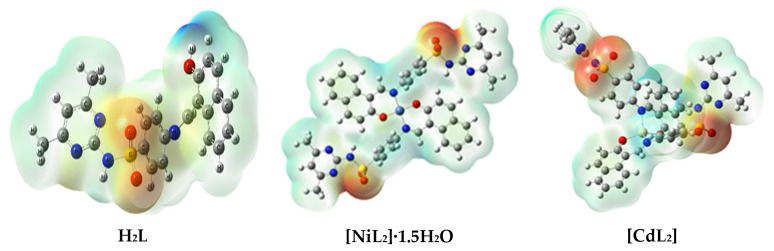
Molecular electrostatic potential (MEP) of H_2_L, [NiL_2_]·1.5H_2_O, and [CdL_2_].

The computed energies of formation, HOMO (eV), LUMO (eV), as well as other chemical parameters connected to HOMO and LUMO energies, were determined for the H_2_L ligand and its metal chelates (Table 7). As reported elsewhere, HOMO energy measures the electron donation character, while LUMO energy measures the electron affinity [32]. Higher stability compared to free ligand is shown by more negative metal chelate formation energy values [33]. The chelating agent’s computed (E_g_) is greater than that of the Ni(II) and Cd(II) chelates, demonstrating its affinity for binding to the Ni(II) and Cd(II) ions [34]. The extraordinarily low dipole moment value of the Ni(II) complex may be caused by the two ligands canceling each other out due to their trans-orientation (Figure 9 and Figure 10). On the other hand, MEP maps (Figure 11) were created to distinguish the electrostatic potential of positive, negative, and neutral zones using the colors blue, red, and green, respectively. The MEP contains positive zones around hydrogen atoms and negative zones over electronegative atoms (oxygen and nitrogen).

### 2.9. Antitumor Activity

**Figure 12:**
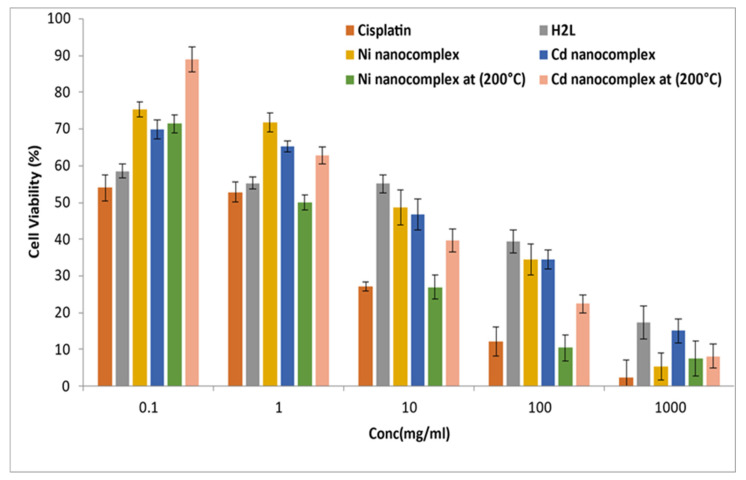
The cell viability of H_2_L and its nano metal chelates versus cis-platin drug.

**Figure 13:**
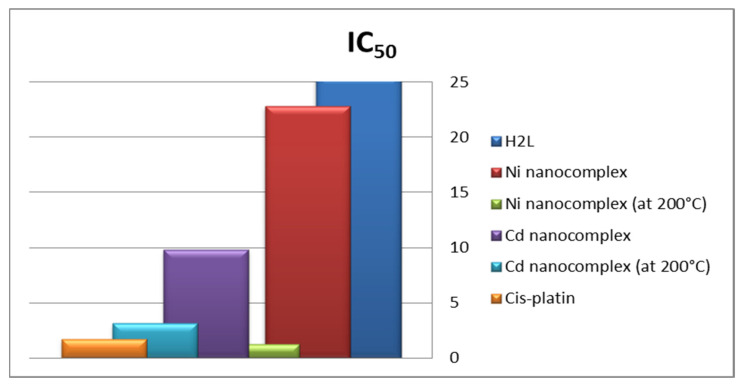
In vitro antitumor activity (IC_50_) of H_2_L and its nano metal chelates (before and after) heating against HepG-2 cells.

The anticancer activity of the ligand H_2_L and its Ni and Cd nano-chelates generated in CS/EtOH (before and after) heating them was examined against the HepG-2 human cancer cell line. Cis-platin (IC_50_ = 1.714 μg/mL) was used as the standard. Compounds with IC50 values of less than 5.00, 5.00–10.00, and 10.00–25.00 μg/mL are categorized as strong, moderate, and weak antitumor agents, respectively [35]. The growth of the HepG-2 cancer cells was generally inhibited by the investigated compounds in a concentration-dependent manner. Moreover, the examined nano metal chelates were more cytotoxic than the comparable free ligand. This could be attributed to the metal’s redox-active center [36]. Interestingly, the nano-sized Ni complex and Cd complex, after heating, displayed strong cytotoxicity compared with other compounds, whereas the Ni nanocomplex after heating exhibited antitumor activity (IC50 = 1.280 g/mL), which was higher than the activity of cis-platin (IC50 = 1.714 g/mL), and the Cd nanocomplex (before heating) displayed moderate antitumor activity (Figure 12 and Figure 13).

### 2.10. In Vitro Antimicrobial Results

**Figure 14:**
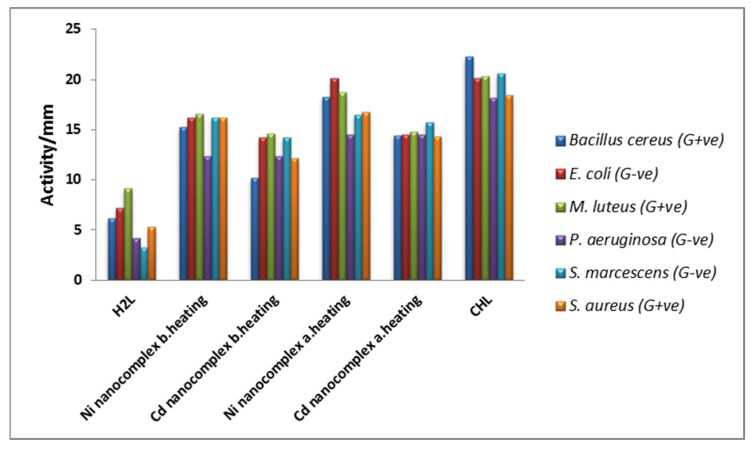
Antibacterial activity of H_2_L and its nanocomplexes.

**Figure 15:**
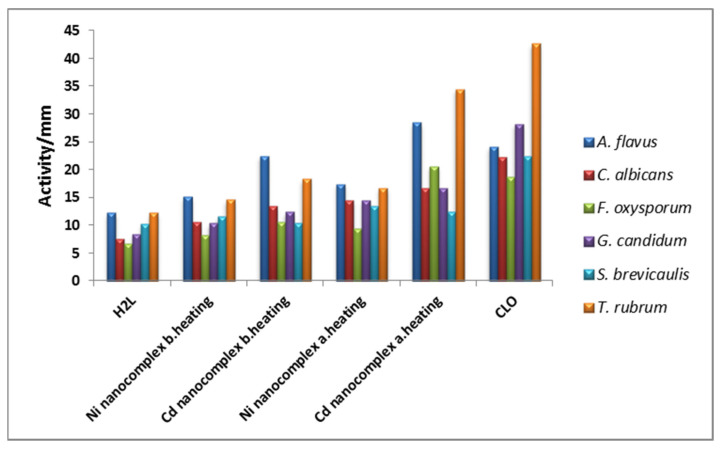
Antifungal activity of H_2_L and its nanocomplexes.

The newly synthesized Schiff base ligand (H_2_L) and its Ni and Cd nanocomplexes (before and after) heating were investigated for their inhibitory effects on the growth of *Bacillus cereus* (*G+ve*), *E. coli* (*G−ve*), *Micrococcus luteus* (*G+ve*)*, Pseudomonas aeruginosa* (*G−ve*), *Serratia marcescens* (*G−ve*)*,* and *Staphylococcus aureus* (*G+ve*) bacteria, and *Aspergillus flavus*, *Candida albicans*, *Fusarium oxysporum*, *Geotrichum candidum*, *Scopulariopsis brevicaulis*, and *Trichophyton rubrum* fungi. The antimicrobial activity was tested using the disk diffusion method; the clear zone of inhibition around each disk was measured (in mm) and compared to the known sensitive drugs: chloramphenicol (CHL) as an antibacterial drug and clotrimazole (CLO) as an antifungal drug. The findings suggest that the nanocomplexes exhibit greater activity as compared to the H_2_L ligand under the same experimental conditions. This would indicate that the chelation could improve the ability of a nanocomplex to cross a cell membrane, which can be described by Tweedy’s chelation theory [37]. From the data in Table 8, the nano-range complexes after heating displayed higher influences on the tested bacteria compared with other compounds, indicating that the decrease in particle size caused by heating increases the activities. In case of antifungal activity, Cd nanocomplexes after heating showed good antifungal results against *A. flavus* and *F. oxysporum* within the inhibition zone, at 28 and 20 mm, which are greater than those of the standard clotrimazole (24 mm) and (18 mm), respectively (Figure 14 and Figure 15).

The growth of bacterial pathogens on each concentration was checked to determine the minimum concentration that inhibits the growth of the organism. It is evident from Table 9 that the MIC value for the Ni nanocomplex after heating was 0.625 mg/mL for *B. cereus*, *E. coli* (*G−ve*), and *Serratia marcescens* (*G−ve*). Furthermore, the Ni nanocomplex after heating demonstrates antifungal activity against the tested fungi, but *Trichophyton rubrum* appeared as the most sensitive fungus among all fungi involved in this study (Table 9).

### 2.11. Molecular Docking Studies

**Figure 16:**
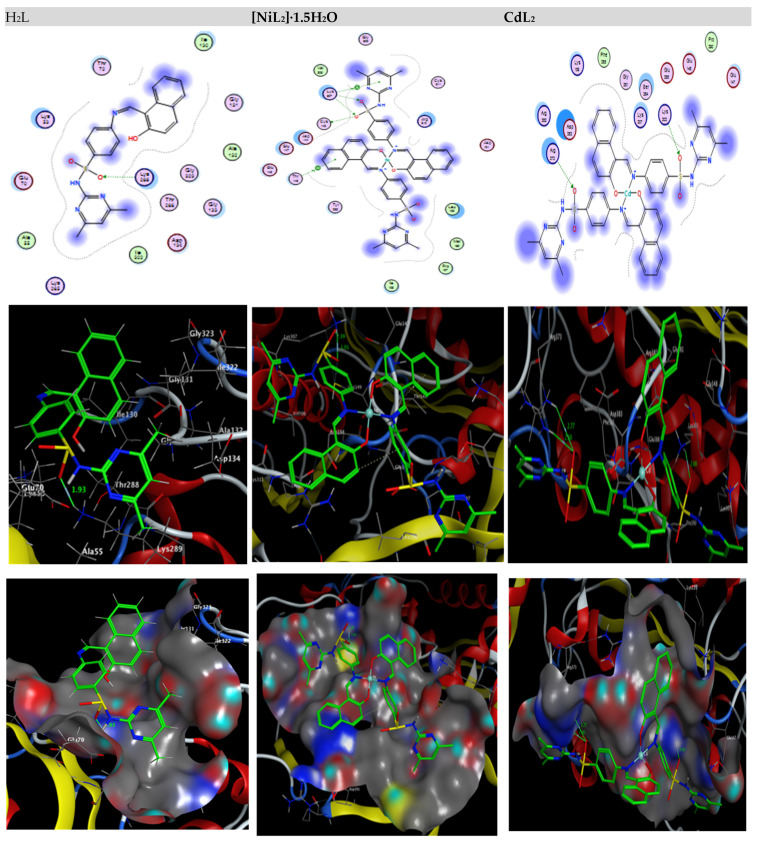
Two-dimensional and three-dimensional plots of the interactions between H_2_L, [NiL_2_]·1.5H_2_O, and CdL_2_ with the (PDB ID: 5A19) protein receptor.

**Figure 17:**
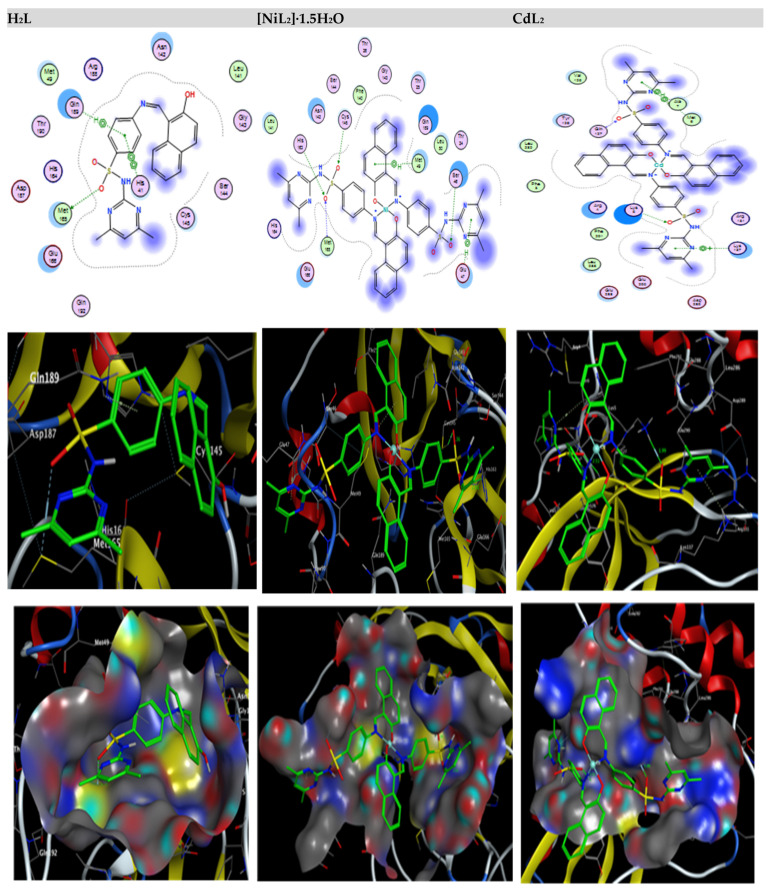
Two-dimensional and three-dimensional plots of the interactions between H_2_L, [NiL_2_]·1.5H_2_O, and CdL_2_ with the (PDB ID: 6lu7) protein receptor.

Molecular docking is a potent method for analyzing the biological activity of target molecules and producing distinguishing structural features for the creation of novel therapeutics. Moreover, it is a common computational technique for determining binding sites with appropriate conformations and estimating binding affinity. To predict the possible binding modes at the active pockets, all prepared compounds were docked with methionine adenosyl-transferases (PDB ID: 5A19) and COVID-19 main protease viral protein (PDB ID: 6lu7) (Figure 16 and Figure 17). The results demonstrated that the ligand’s dominant interaction force was the H-acceptor, although other binding interactions, such as the H donor, also took place in complexes. A stronger interaction between the investigated compounds and receptors reflects more negative energy. As a result, the interaction ability towards the receptor was arranged as follows: in the case of (PDB ID: 5A19), NiL_2_ > CdL_2_ > H_2_L, whereas in the case of (PDB ID: 6lu7), CdL_2_ > NiL_2_ > H_2_L (Table 10 and Table 11).

## 3. Materials and Methods

### 3.1. Materials and Equipment

In-depth information about the chemicals, tools, and processes used for the structural verification and applications can be found in Section S1.

### 3.2. Procedure of Schiff Base Synthesis

**Scheme 1:**
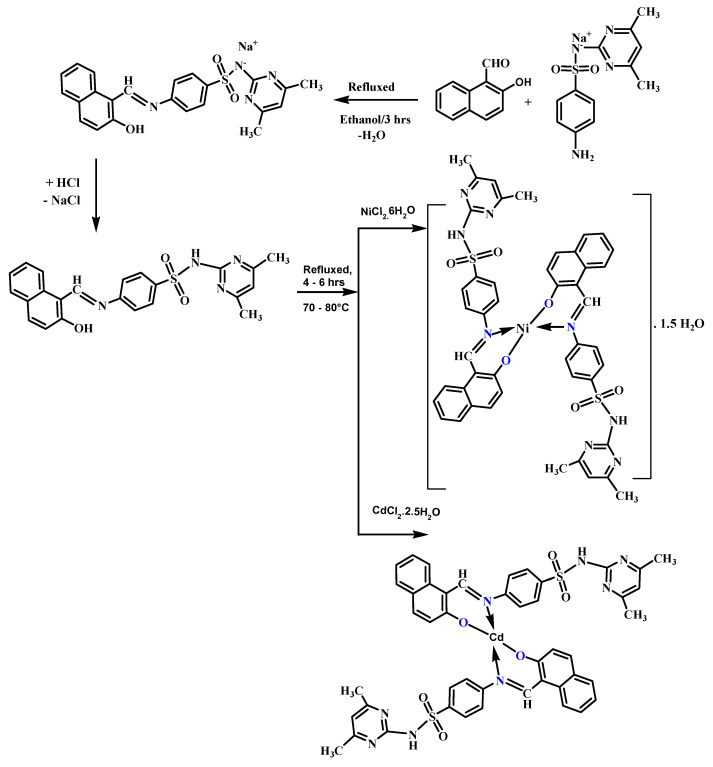
General route for the synthesis of the Schiff base ligand (H_2_L) and its metal complexes.

The tested H_2_L ligand was prepared by adding a solution of (2.7833 g, 10 mmol) Sulfamethazine sodium salt to a solution of 2-hydroxy-1-naphthaldehyde (1.7218 g, 10 mmol) in 20 mL EtOH. The reaction mixture was left under reflux for 4 h at 70 °C. The resulting yellow solid was then mixed with dilute hydrochloric acid (5%) to form a neutral solution. The yellow precipitate was recrystallized from EtOH and then dried in a vacuum (Scheme 1).

### 3.3. Procedure of Metal Complex Synthesis

The metal chelates were prepared by refluxing a mixture of the H_2_L ligand in 20 mL hot ethanol with a solution of NiCl_2_·6H_2_O or CdCl_2_·2.5H_2_O in a 1:1 molar ratio. The reaction mixtures were kept under reflux for 4–6 h at 70 °C, filtered, washed with ethanol, and dried at room temperature (Scheme 1).

### 3.4. Procedure of Nanocomplex Synthesis

Nano domain compounds of Ni(II) and Cd(II) were created in a 1:1 molar ratio by adding *Coriandrum sativum* (CS) media extract in 20% ethanol, as explained previously in our publications [32,38].

### 3.5. DFT Studies

The Schiff base ligand and its Ni(II) and Cd(II) chelates were optimized with the Gaussian 09 program [39], by implementing the LANL2DZ basis set for Ni and Cd metals, while the 6-311++G basis set was used for the rest of the atoms [40].

### 3.6. Cytotoxicity Assay

The antitumor property of the examined compounds was measured on a microplate reader (Sunrise, Tecan, Inc., East Lyme, CT, USA) using 490 nm filters against the Hepatocellular carcinoma cell line, HepG-2 cells (ATCC No. HB-8064), and compared to the cis-platin (cis-diamminedichloroplatinium) as reference drug. The experiments were conducted in the tissue culture section of Al-Azhar University’s Regional Institute for Mycology and Biotechnology in Cairo, Egypt. The IC_50_ values were calculated using the graphed prism version [41].

### 3.7. Anti-Pathogenic Activities

The prepared compounds were screened against *Bacillus cereus* (*G+ve*), *E. coli* (*G−ve*), *Micrococcus luteus* (*G+ve*), *Pseudomonas aeruginosa* (*G−ve*), *Serratia marcescens* (*G−ve*), and *Staphylococcus aureus* (*G+ve*) bacteria, and *Aspergillus flavus*, *Candida albicans*, *Fusarium oxysporum*, *Geotrichum candidum*, *Scopulariopsis brevicaulis*, and *Trichophyton rubrum* fungi [42].

### 3.8. Molecular Docking (MD)

Molecular docking studies were performed using the MOE2019 software. The output file of the Gaussian 09 software was used to convert the structures of the H_2_L ligand and its complexes into a PDB format file. The crystal structures of the target proteins for (PDB ID: 5A19) in liver cancer [43] and COVID-19 main protease (PDB ID: 6lu7) [44] were downloaded from the Protein Data Bank (https://www.rcsb.org accessed on 1 January 2020). For protein preparation, the bounded water molecules, the co-crystallized ligand, and the other cofactors were removed. In order to assign the charges and the parameters, an MMFF94x force field was used. The MOE site finder was used to produce the most probable protein binding sites. The energy of the protein molecules and the prepared compounds was reduced utilizing the energy minimization algorithm of the Molecular Operating Environment (MOE2019 software). The binding affinity between the protein and the generated compounds was determined through analyzing the binding energies of the top ten poses. By comparing the values of the binding-free energy and hydrogen bond lengths, the best scoring binding affinity could be determined. The estimated hydrogen bond lengths should not exceed 3.5 Å.

## 4. Conclusions

In this study, two types of nano-sized complexes have been synthesized that are characterized by various physicochemical and spectral analyses. The ligand H_2_L acts as a bidentate chelate by coordinating to the metal ions through NO donor atoms, forming square planar and tetrahedral complexes. Furthermore, the prepared complexes’ kinetic and thermodynamic properties are evaluated. The particles of the complexes under investigation were sub-nanoscale, as revealed by the XRD and TEM results. The findings reveal a decrease in the size of nano metal chelates after heating. The DFT calculations of the prepared compounds confirm the experimental results. The antimicrobial activities of the ligand, H_2_L, and its nano metal chelates showed that the metal ion in the nanocomposites enhanced the antimicrobial activities in comparison to the free ligand. Also, all compounds under examination demonstrated a reduction in HEPG-2 cell growth, and heating the nanocomplexes increased their cytotoxic effects. The Ni nanocomplex exhibited substantial antitumor activity (IC50 = 1.280 g/mL), which was higher than the activity of cis-platin (IC50 = 1.714 g/mL). Additionally, a molecular docking study suggests that [NiL_2_]·1.5H_2_O and CdL_2_ have the most activity with (PDB ID: 5A19) and (PDB ID: 6lu7) cell-proteins, respectively. It is worth mentioning that there is a limitation for using the investigated compounds as drugs due to their lack of stability. Also, there is a need for further investigation of the toxicity of the prepared complexes towards normal cancer cells.

## Figures and Tables

**Table 1:** Analytical and physical data of the compounds studied.

Compound	Chemical Formula (M.Wt.)	Color	M.P. °C	Ω_m_ (ohm^−1^ cm^2^ mol^−1^)	Analysis: Found (calc.), %			
					%C	%H	%N	%S
H_2_L	C_23_H_20_N_4_O_3_S (432.50)	Yellow	185	0.7	63.69 (63.87)	4.48 (4.66)	12.73 (12.95)	7.52 (7.41)
[NiL_2_]·1.5H_2_O	C_46_H_41_N_8_NiO_7.5_S_2_ (948.70)	Black	>300	6	58.37 (58.24)	4.45 (4.36)	11.63 (11.81)	6.82 (6.76)
CdL_2_	C_46_H_38_CdN_8_O_6_S_2_ (975.39)	Reddish brown	>300	12	56.89 (56.64)	3.75 (3.93)	11.33 (11.49)	6.68 (6.57)

**Table 2:** Thermoanalytical results and the particle size of Ni and Cd nanocomplexes before heating.

Nanocomposite	Media	Temperature Range (°C)	Found (%)	Particle Size (nm)
Ni nanocomplex	*C.S*	48–110 110–260 260–618 618–1000	12.89 20.25 29.40 16.14	21.39 17.06 12.05 10.11
Cd nanocomplex		22–220 220–524 524–1000	5.68 29.51 42.70	31.96 22.53 12.90

**Table 3:** Thermoanalytical results and the particle size of Ni and Cd nanocomplexes after heating.

Nanocomposite	Media	Temperature Range (°C)	Found (%)	Particle Size (nm)
Ni nanocomplex	*C.S*	29–204 204–550 550–1000	14.52 34.10 20.89	3.94 2.60 2.05
Cd nanocomplex		29–176 176–566 566–1000	9.53 28.90 44.94	2.78 1.97 1.08

**Table 4:** The kinetic and thermodynamic data of the thermal decomposition of complexes.

Compound	Step	r^2^	n	Z, (min^−1^)	E*	∆S*	∆H*	∆G*
[NiL_2_]·1.5H_2_O	1	0.9953	1	36.2 × 10^4^	34.68	−153.17	31.72	86.18
	2	0.9996	1	2.4 × 10^13^	157.00	−13.34	152.00	159.15
	3	0.9957	2	5.2 × 10^8^	137.12	−85.74	130.82	195.72
	4	0.9822	2	1.2 × 10^52^	1008.19	741.34	999.70	244.38
[CdL_2_]	1	0.9783	2	1.6 × 10^3^	118.2	−245.43	113.44	255.04
	2	0.9979	2	7.7 × 10^16^	55.82	70.44	49.77	−1.43
	3	0.9993	2	3.0 × 10^55^	1142.16	806.7	1133.30	274.66

r^2^ = correlation value of regression, n = order of the decomposition reaction, Z = pre-exponential factor in min^−1^; E*, ∆H*, and ∆G* are in kJ mol^−1^, ∆S* in JKmol^−1^.

**Table 5:** UV-visible spectral data of H_2_L and its micro- and nanocomplexes.

Compound	Spectral Bands (nm)	Wavenumber (cm^−1^)	ε_max_ (L.mol^−1^ cm^−1^)	Band Assignment	Geometry
**H_2_L**	386 445 468 539	25,906 22,471 21,367 18,552	12,610 9940 9270 1169	π→π* n→π* n→π* CT	---------
**[NiL_2_]·1.5H_2_O**	365 408 542	27,397 24,509 18,450	6491 5086 1132	n→π* ^1^A_1_g→^1^B_1_g ^1^A_1_g→^1^A_2_g	Square planar
**CdL_2_**	456 525	21,929 19,047	5138 3276	n→π* CT	Tetrahedral
**Ni nanocomplex**	363 394 535	27,548 25,380 18,691	11,050 8202 1782	n→π* ^1^A_1_g→^1^B_1_g ^1^A_1_g→^1^A_2_g	Square planar
**Cd nanocomplex**	406 513	24,630 19,493	11,440 6713	n→π* CT	Tetrahedral

**Table 6:** Important optimized bond lengths (Å) and bond angles (o) of the prepared [NiL_2_]·1.5H_2_O and [CdL_2_] complexes.

[NiL_2_]·1.5H_2_O				
Bond	Bond Length (Å) Complex	Bond	Bond Length (Å) H_2_L Complex	
Ni-O1	1.855	N1-----O1	3.715	2.69064
Ni-O4	1.855	N5-----O4	3.715	2.69061
Ni-N1	1.92523			
Ni-N5	1.92523			
Angle	Angle (°) Complex	Angle	Angle (°) Complex	
N1-Ni-O1	90.71470	O1-Ni-N5	89.28729	
N1-Ni-N5	179.99223	O1-Ni-O4	179.99581	
N1-Ni-O4	89.28483	O4-Ni-N5	90.71317	
		O1-N1-O4-N5	0.008 *	
	**[CdL_2_]**			
Bond	Bond length (Å) Complex	Bond	Bond length (Å) H_2_L Complex	
Cd-O1	2.153	N1-----O1	3.715	2.950
Cd-O4	2.154	N5-----O4	3.715	2.948
Cd-N1	2.27750			
Cd-N5	2.28187			
Angle	Angle (°) Complex	Angle	Angle (°) Complex	
N1-Cd-O1	83.45095	O1-Cd-N5	109.65028	
N1-Cd-N5	138.91524	O1-Cd-O4	142.85925	
N1-Cd-O4	110.01703	O4-Cd-N5	83.24996	

* dihedral angle.

**Table 7:** Computed chemical parameters of the ligand and its complexes.

Property		H_2_L	[NiL_2_]·1.5H_2_O	[CdL_2_]
Total energy E (a.u.)		−1731.338	−3630.46	−3509.19
HOMO (eV)		−5.8499	−5.8847	−6.0575
LUMO (eV)		−1.6191	−2.4558	−2.6503
E_g_ = E_LUMO_ − E_HOMO_ (eV)		4.2308	3.4289	3.4072
Dipole moment (Debye)		6.1304	0.002583	7.9638
Ionization potential	I = −E_HOMO_	5.8499	5.8847	6.0575
Electron affinity	A = −E_LUMO_	1.6191	2.4558	2.6503
Electronegativity	χ = (I +A)/2	3.7345	4.17025	4.3539
Chemical hardness	η = (I − A)/2	2.1154	1.7144	1.7036
Chemical softness	S = 1/2η	0.2364	0.8572	0.8518
Chemical potential	μ = −χ	−3.7345	−4.1702	−4.3539
Electrophilicity	ω = μ^2^/2η	3.2964	5.0718	5.5636

**Table 8:** Antibacterial and antifungal activity (inhibition zone in mm) of chemical compounds.

Bacteria	Compound					
	H_2_L	Ni Nanocomplex B. H. *	Cd Nanocomplex B. H. *	Ni Nanocomplex A. H. *	Cd Nanocomplex A. H. *	CHL
*Bacillus cereus* (*G+ve*)	6.15 ± 0.11	15.25 ± 0.14	10.15 ± 0.08	18.25 ± 0.09	14.40 ± 0.07	22.25 ± 0.13
*E. coli* (*G−ve*)	7.20 ± 0.08	16.18 ± 0.15	14.25. ± 0.05	20.15 ± 0.16	14.50 ± 0.05	20.15 ± 0.04
*Micrococcus luteus* (*G+ve*)	9.10 ± 0.12	16.55 ± 0.13	14.60 ± 0.03	18.75 ± 0.09	14.75 ± 0.11	20.35 ± 0.15
*Pseudomonas aeruginosa* (*G−ve*)	4.15 ± 0.15	12.35 ± 0.08	12.35 ± 0.12	14.45 ± 0.05	14.45 ± 0.08	18.15 ± 0.05
*Serratia marcescens* (*G−ve*)	3.25 ± 0.04	16.20 ± 0.07	14.25 ± 0.03	16.50 ± 0.13	15.75 ± 0.11	20.55 ± 0.14
*Staphylococcus aureus* (*G+ve*)	5.25 ± 0.06	16.15 ± 0.09	12.10 ± 0.04	16.70 ± 0.12	14.30 ± 0.13	18.45 ± 0.06
**Fungi**	**Compound**					
	**H_2_L**	**Ni Nanocomplex** B. H. *	**Cd Nanocomplex** B. H. *	**Ni Nanocomplex** A. H. *	**Cd Nanocomplex** A. H. *	**CLO**
*Aspergillus flavus*	12.25 ± 0.15	15.13 ± 0.07	22.50 ± 0.16	17.45 ± 0.14	28.50 ± 0.09	24.20 ± 0.14
*Candida albicans*	7.55 ± 0.09	10.60 ± 0.05	13.45 ± 0.15	14.50 ± 0.18	16.75 ± 0.07	22.35 ± 0.15
*Fusarium oxysporum*	6.65 ± 0.12	8.25 ± 0.13	10.55 ± 0.10	9.45 ± 0.12	20.65 ± 0.06	18.75 ± 0.16
*Geotrichum candidum*	8.45 ± 0.13	10.40 ± 0.19	12.40 ± 0.10	14.45 ± 0.07	16.75 ± 0.09	28.25 ± 0.18
*Scopulariopsis brevicaulis*	10.30 ± 0.07	11.55 ± 0.09	10.50 ± 0.05	13.45 ± 0.14	12.40 ± 0.10	22.45 ± 0.15
*Trichophyton rubrum*	12.25 ± 0.09	14.65 ± 0.12	18.35 ± 0.08	16.75 ± 0.06	34.55 ± 0.11	42.75 ± 0.09

B. H. * = before heating; A. H. * = after heating.

**Table 9:** Antibacterial and antifungal activity (inhibition zone and MIC) of chemical compounds (mg/mL).

Bacteria	Compound					
	H_2_L	Ni Nanocomplex B. H. *	Cd Nanocomplex B. H. *	Ni Nanocomplex A. H. *	Cd Nanocomplex A. H. *	CHL
*Bacillus cereus* (*G+ve*)	3.15 (3.25)	8.45 (1.25)	8.25 (2.5)	8.50 (0.625)	8.15 (2.5)	10.25 (0.078)
*E. coli* (*G-ve*)	5.20 (4.25)	10.75 (1.25)	7.60 (2.5)	8.25 (0.625)	10.25 (2.5)	10.15 (0.156)
*Micrococcus luteus* (*G+ve*)	7.45 (3.75)	10.25 (2.5)	8.50 (5)	14.45 (2.5)	8.45 (1.25)	10.50 (0.625)
*Pseudomonas aeruginosa* (*G-ve*)	2.15 (2.50)	8.50 (5)	8.75 (5)	10.55 (1.25)	8.15 (2.5)	10.75 (0.156)
*Serratia marcescens* (*G-ve*)	2.25 (3.25)	6.40 (1.25)	8.40 (2.5)	6.35 (0.625)	10.30 (2.5)	10.25 (0.625)
*Staphylococcus aureus* (*G+ve*)	3.25 ± (4.75)	6.25 (0.625)	6.50 (2.5)	8.15 (2.5)	6.15 (1.25)	10.15 (0.08)
**Fungi**	**Compound**					
	**H_2_L**	**Ni Nanocomplex** B. H. *	**Cd Nanocomplex** B. H. *	**Ni Nanocomplex** A. H. *	**Cd Nanocomplex** A. H. *	**CLO**
*Aspergillus flavus*	8.15 (17)	10.45 (12.75)	18.15 (20)	11.15 (12)	8.45 (5)	10.45 (0.039)
*Candida albicans*	6.20 (16)	8.45 (14.50)	14.20 (20)	9.40 (13)	8.55 (2.5)	8.60 (0.312)
*Fusarium oxysporum*	4.85 (21)	10.55 (20)	10.75 (20)	9.11 (13.40)	17.35 (20)	12.25 (0.312)
*Geotrichum candidum*	5.45 (18)	7.45 (3.5)	7.25 (21)	8.35 (2.5)	9.50 (14)	10.15 (0.156)
*Scopulariopsis brevicaulis*	7.40 (19)	9.50 (21)	10.50 (20)	16.75 (20)	8.35 (5)	8.75 (0.078)
*Trichophyton rubrum*	10.25 (14)	11.35 (4.5)	27.24 (20)	18.25 (2.5)	10.15 (5)	22.30 (0.025)

B. H. * = before heating; A. H. * = after heating; numbers outside parentheses reveal the inhibition zone in (mm); numbers in parentheses illustrate the MIC in (mg/mL) of the tested compounds.

**Table 10:** Docking interaction data for the ligand (H_2_L) and its complexes towards liver cancer protein (PDB ID: 5A19).

	Receptor	Interaction	Distance (Å) *	E (kcal/mol)
H_2_L				
O 12	NZ LYS 289	H-acceptor	2.82 (1.93)	−26.0
[NiL_2_]·1.5H_2_O				
O 52	NZ LYS 307	H-acceptor	2.90 (1.91)	−29.4
O 53	NZ LYS 307	H-acceptor	3.06 (2.39)	−3.0
O 52	SG CYS 149	H-donor	3.48 (3.00)	−2.9
6-ring	CA THR 146	pi-H	4.00	−1.3
6-ring	CA LYS 307	pi-H	4.39	−0.9
CdL_2_				
O 20	NE ARG 373	H-acceptor	3.65 (2.77)	−0.7
O 20	NH2 ARG 373	H-acceptor	3.51 (2.60)	−2.5
O 52	NZ LYS 303	H-acceptor	2.91 (2.00)	−24.8

* The lengths of H-bonds are in brackets.

**Table 11:** Docking interaction data for the ligand (H_2_L) and its complexes towards COVID-19 main protease viral protein (PDB ID: 6lu7).

	Receptor	Interaction	Distance (Å) *	E (kcal/mol)
H_2_L				
O 12	SD MET 165	H-donor	3.27 (2.07)	−6.9
6-ring	NE2 GLN 189	pi-H	4.67	−1.1
6-ring	5-ring HIS 41	pi-pi	3.98	−0.1
[NiL_2_]·1.5H_2_O				
O 21	CB SER 46	H-acceptor	3.49 (2.51)	−0.8
O 52	SG CYS 145	H-acceptor	3.66 (2.36)	−2.0
O 53	NE2 HIS 163	H-acceptor	3.36 (2.61)	−1.2
O 53	CA MET 165	H-acceptor	3.25 (2.44)	−0.9
6-ring	CA GLU 47	pi-H	4.13	−1.5
6-ring	CE MET 49	pi-H	3.74	−0.5
CdL_2_				
O 20	N GLN 127	H-acceptor	3.49 (2.52)	−2.9
O 52	NZ LYS 5	H-acceptor	2.94(1.99)	−26.4
6-ring	CA MET 6	pi-H	4.24	−0.9
6-ring	N ALA 7	pi-H	3.62	−3.1
6-ring	NZ LYS 137	pi-cation	4.42	−0.5

* The lengths of H-bonds are in brackets.

## Data Availability

The raw/processed data generated in this work are available upon request from the corresponding author.

## Supplementary Materials

The following supporting information can be downloaded at: https://www.mdpi.com/article/10.3390/ijms241814259/s1.
