# Supplementary material for: Development of Novel Nano-Sized Imine Complexes Using Coriandrum sativum Extract: Structural Elucidation, Non-Isothermal Kinetic Study, Theoretical Investigation and Pharmaceutical Applications
Source: Int J Mol Sci. 2023 Sep 19;24(18):14259. doi: 10.3390/ijms241814259 (PMC10531680; doi:10.3390/ijms241814259)
Supplement: Supplementary file 1 [file ijms-24-14259-s001.zip › ijms-2580735-supplementary.pdf]

Development of novel nano sized imine complexes using Coriandrum sativum extract: Structural elucidation, non-isothermal kinetic study, theoretical investigation and pharmaceutical applications

Shimaa Hosny [1,*], Randa F. Abd El-Baki [1], Zeinab H. Abd El-Wahab [2] and Gamal A. Gouda [3], Mohammed S. Saddik[4], Ateyatallah Aljuhani[5], Ahmed M. Abu-Dief[5,6,*]

[1] Department of Chemistry, Faculty of Science, New Valley University, Alkharga 72511, Egypt; shimaahosny@sci.nvu.edu.eg; randafouad_1974@sci.nvu.edu.eg
[2] Department of Chemistry, Faculty of Science (Girl's), Al-Azhar University, Cairo, 11754, Egypt; zhabdelwahab@azhar.edu.eg
[3] Department of Chemistry, Faculty of Science, Al-Azhar University, Assiut Branch, 71524, Assiut, Egypt; ggouda73@azhar.edu.eg
[4]Department of Pharmaceutics and Clinical Pharmacy, Faculty of Pharmacy, Sohag University, P.O. Box 82524, Sohag 82524, Egypt, mohammed.sherif@pharm.sohag.edu.eg

[5] Chemistry Department, College of Science, Taibah University, Madinah 42353, Saudi Arabia amamohammed@taibahu.edu.sa (Ahmed M. Abu-Dief); assjuhani@taibahu.edu.sa (Ateyatallah Aljuhani)
[6] Chemistry Department, Faculty of Science, Sohag University, Sohag 82524, Egypt
* Correspondence: shimaahosny@sci.nvu.edu.eg (Shimaa Hosny) and amamohammed@taibahu.edu.sa (Ahmed M. Abu-Dief)

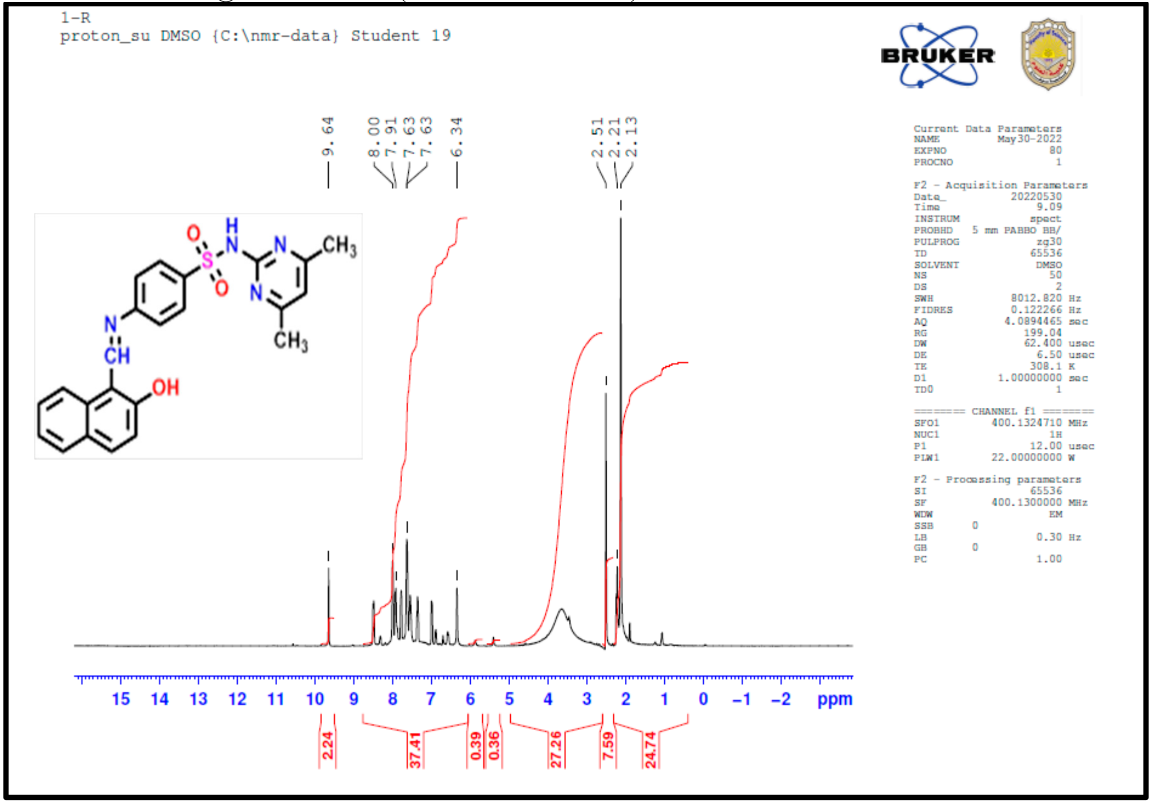

Figure S1. ^{1}H NMR spectrum of H_2L in DMSO

Table S1 Essential vibrational frequencies (cm^{-1}) of H_2L and its micro-complexes

IR bands (cm^{-1})								
**No**	**Compound**	**υ(C=N)**	**υ(O-H); υ (NH)**	**$υ_{as}(SO_2)$**	**$υ_{sy}(SO_2)$**	**$δ(H_2O)$**	**υ(M-O)**	**υ(M-N)**
**I**	H_2L	1633	3200;3457	1384	1156	-----	-----	-----
**II**	$[NiL_2].1.5H_2O$	1602	;3453	1384	1152	970	585	549
**III**	CdL_2	1624	;3458	1384	1157	-----	566	544

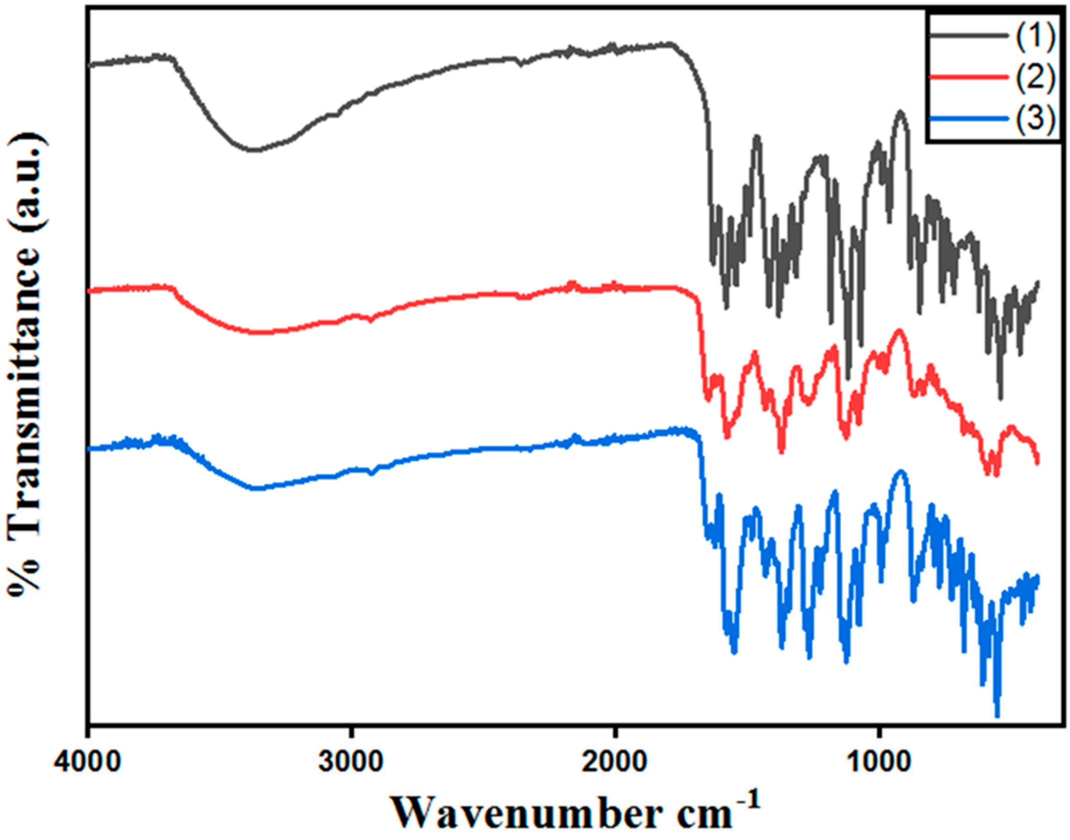

Figure S2. FT-IR spectra of H_2L(1), Ni (II) complex(2) and Cd(II) complex(3)

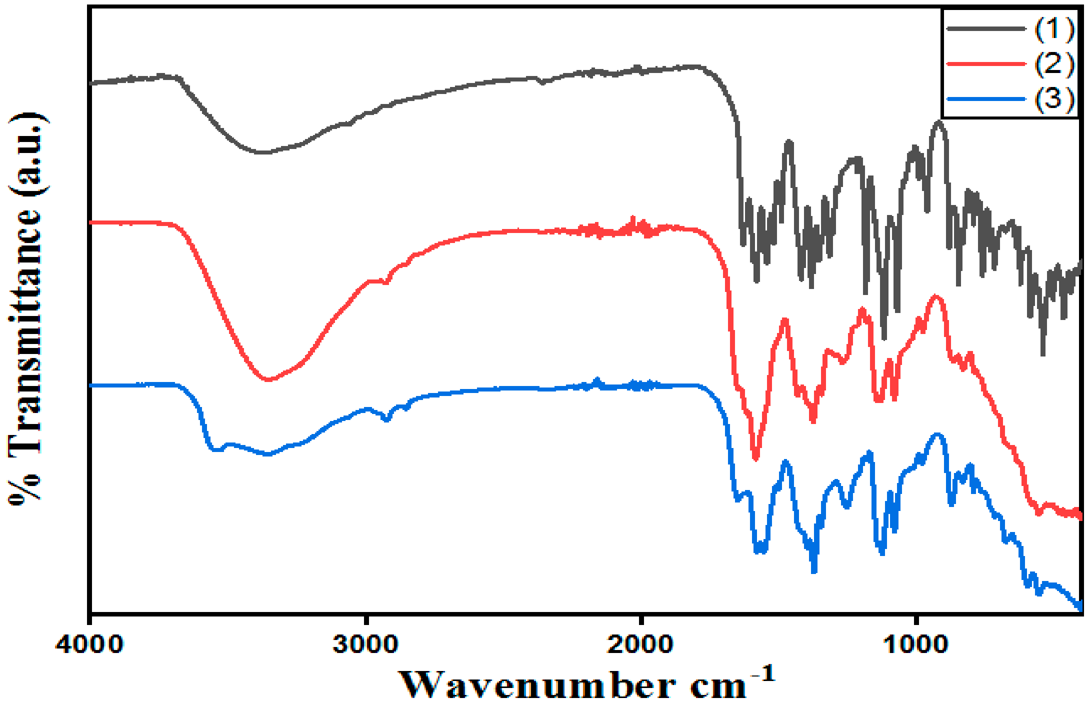

Figure S3. FT-IR spectra of H_2L(1), Ni nano complex (2) and Cd nano complex(3)

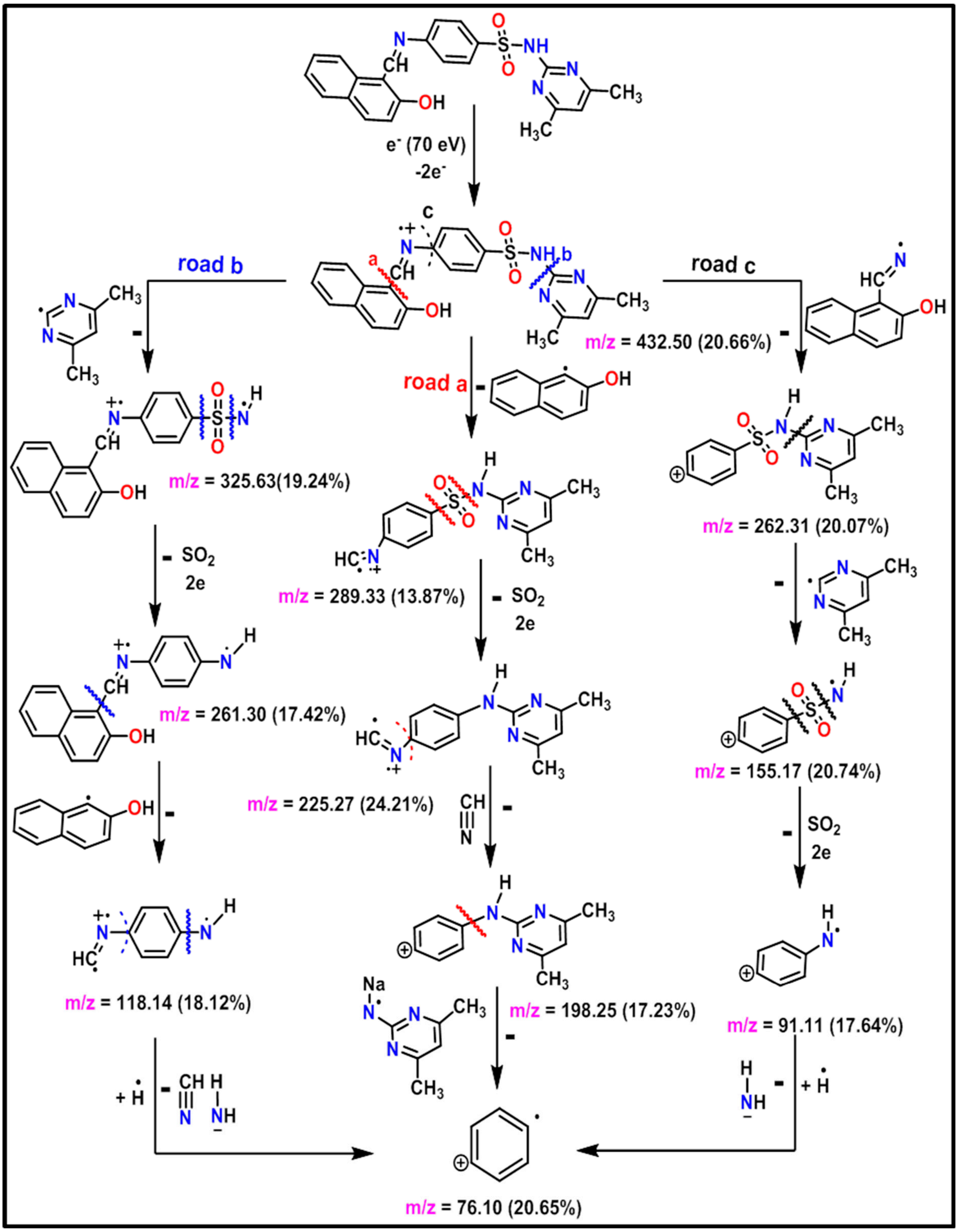

Scheme S1 Suggested MS fragmentation pattern of Schiff base ligand

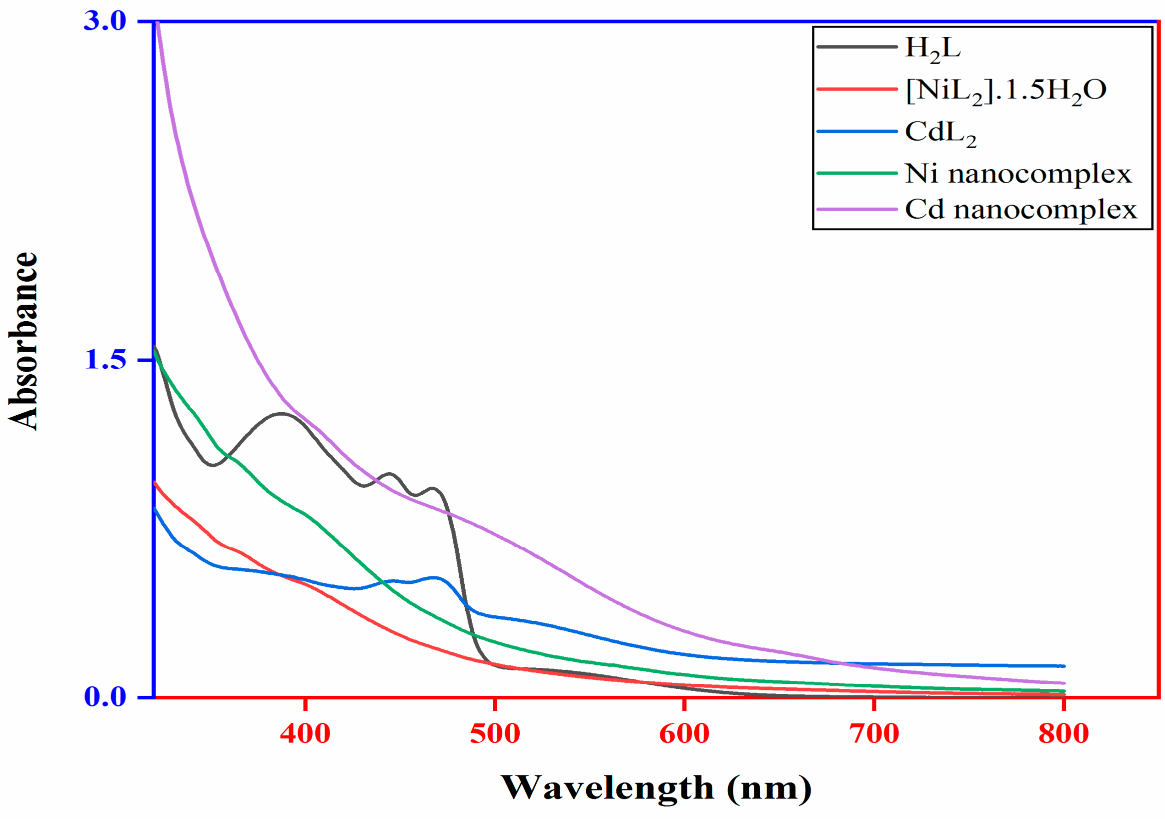

Figure S4. UV-Vis absorption spectra in $I0^{-4}$ DMF solution

Section S1: Materials and equipment's

All chemicals used were obtained from Aldrich or BDH. They included 2-Hydroxy-1-naphthaldehyde, sulfamethazine, $NiCl_2.6H_2O$ or $CdCl_2.2.5H_2O$ and LiOH. The solvents, such as absolute ethyl alcohol and Dimethylformamide (DMF) were used as received without pretreatment.

The characterization of ligand H_2L and their corresponding micro/nano Ni(II), and Cd(II) complexes was carried out utilizing the various spectroscopic techniques: Elemental analyses (C, H, Cl, and N) were performed by Micro analytical unit of the Cairo University, Egypt. ^1H NMR spectra in deuterated dimethyl sulfoxide d_6-DMSO were recorded using a 300 MHz Varian NMR spectrometer. The chemical shift was measured relative to the solvent peak. Mass spectrum for ligand and metal complexes was run on Shimadzu-QP 2010 plus Mass Spectrometer, Microanalytical Laboratory, Faculty of Science, Cairo University, Egypt. The Fourier transform infrared absorption spectra of solid ligand and micro/nano complexes was performed by Micro analytical unit at Cairo University, Egypt, in the wavelength range (4000-400 cm^{-1}) using KBr discs using a Nenexeus-Nicolidite -640-MSA FT-IR infrared

spectrophotometer. The electronic spectra (ultraviolet/visible) were recorded using a Perkin Elmer Lambada 330 spectrophotometer. The electronic spectra of Dimethyl Formamide (DMF) solution of both the ligand and micro/nano complexes were recorded in 1cm quartz cells. The molar conductivity measurements were determined in N, N′ Dimethyl- formamide (DMF) solution at (10^{-3}M) using a tacussel conductimeter type CD6N.

Thermal Analyzer with a heating rate of 10 ^{0}C/min in a nitrogen atmosphere with a following rate of 20 mL/min in the ambient temperature range up to 800 ^{0}C using platinum crucibles. X-ray powder diffraction analyses of solid samples were measured using APD 2000 PROModel GNR-X-ray Diffractometer at (Central lab., Tanta University, Egypt). X-ray diffractograms give computer control formally finished by PHILIPS®MPDX′PERT X-ray diffractometer ready with Cu radiation CuKα (λ=1.540 56 Å). The x′pertdiffractometer has the Bragg-Brentano geometry. The x-ray tube was a copper tube operating at 40 KV and 30 mA. The scanning range (2θ) was 5–90° with a step size of 0.050° and a counting time of 2 s/step. Quartz was used as the standard material to accurate for the instrumental expansion. This identification of the nano complexes was done by a known method. From the fit identified Scherrer formula, the average crystallite size (D) is $D = (K\lambda / \beta \cos \theta)$ Where: λ is the X- ray wavelength in the nanometer, K is a factor related to crystallite shape, with a value of about 0.9 and ß is the peak width at half maximum height. The value of ß in the 2θ axis of diffraction shape must be in radians. The θ is the Bragg angle and can be in radians since the Cos θ compatible with the same number. The surface morphological and particle size study was recorded by TEM micrographs were executed using JEOL JEM-1200 EX II, Japan at 60–70 kV.
